# Unravelling the asymmetric effects of procurement practices on firm performance: A complexity theory approach to complementing fsQCA with NCA

**DOI:** 10.1016/j.heliyon.2024.e25230

**Published:** 2024-01-29

**Authors:** Innocent Senyo Kwasi Acquah

**Affiliations:** a Department of Marketing and Supply Chain Management, School of Business, University of Cape Coast, Ghana; b Faculty of Management Sciences, Durban University of Technology, Durban, South Africa

**Keywords:** Procurement planning, Supplier partnership, Contract management, Compliance, Complexity theory, Contrarian case analysis, Fuzzy-set qualitative comparative analysis (fsQCA), Necessary condition analysis (NCA)

## Abstract

Current economic upheavals and supply chain uncertainty have threatened the profitability and sustainability of business organisations. Procurement has proved to be one of the strategies for enhancing firm performance without necessarily increasing revenue with its attendant increase in costs. However, rather than investigating the complex asymmetric relationship between procurement practices and firm performance (which this study advocates), past research engaged in a symmetric evaluation of the relationship between the phenomena. Accordingly, this study, using complexity theory, employs fsQCA and NCA on a sample of 150 respondents from private universities in Ghana to (a) identify different combinations of procurement practices, namely procurement planning, supplier partnership, contract management, and compliance, that lead to firm performance and (b) explore the necessity of these procurement practices (in kind and degree) for firm performance. Whereas the findings from fsQCA reveal three distinct combinations of procurement practices for high firm performance and further suggest that none of the procurement practices was necessary for firm performance, the NCA results suggest that two out of the four procurement practices investigated are necessary for firm performance and hence must be present in the causal recipes produced by fsQCA to guarantee that they lead to firm performance. The study offers pathways to firm performance through procurement practices and demonstrates how to complement fsQCA with NCA to ensure that causal recipes produced by fsQCA can produce the outcome.

## 1 Introduction

Current economic pressures and uncertainty of supply chains have forced organisations to adopt cost-minimisation strategies, which have resulted in growing attention toward procurement strategy as an avenue to navigate these turbulent times [[1], [2], [3], [4]]. Procurement has proved to be an appropriate strategy for achieving competitive advantage and provides enormous potential for achieving value for money and high profitability without an increase in prices [5]. Procurement as a conduit for achieving competitive advantage stems from the fact that it constitutes about 70 % of the firm's total expenditure. However, to be successful amid the current economic pressures requires investment in strategies and practices that can produce competitive advantage through the reduction in cost besides the efficient use of resources [6]. Accordingly, the need to achieve value for money is much more relevant now, than ever before [1,7,8].

Historically, procurement research has focused on how each procurement practice symmetrically predicts firm performance [6,7,9,10]. This ignores complexity theory and the fact that procurement is a complex social phenomenon that needs to be studied from a complexity perspective through asymmetric rather than symmetric modelling.

During the past decades, intense competition among firms has dramatically changed how firms compete, bringing forth new ways of improving firm performance and increasing profits without necessarily increasing sales [[1], [2], [3], [4]]. Further, past literature suggests that procurement is a significant driver of firm performance according to Refs. [2,3], thereby providing firms with new ways of competing and achieving value for money [4,11,12]. Hence, business researchers suggest that firms should initiate and design policies that promote the proper implementation of appropriate procurement practices for enhanced organisational performance [13,14]. Nevertheless, comprehending procurement as a complex construct comprises several practices, such as procurement planning, supplier partnership, contract management, and compliance [14,15] has been a challenge, and the complex and heterogeneous relationships between procurement practices and firm performance have exacerbated their asymmetric patterns [16]. Owing to such complexity, scholars have made a case for the asymmetric modelling of the nexus between procurement and firm performance by using novel approaches that can identify these complex interactions [[17], [18], [19]].

Existing research on the effect of procurement practices on firm performance focused mainly on symmetric approaches that sought to ascertain the net effects of these practices on firm performance and failed to ascertain the configurational effects of these procurement practices on firm performance. However, these symmetric approaches do not allow for unearthing the possible asymmetric relationships that might be present between procurement practices and firm performance, which might either stem from (a) combinations of procurement practices that enhance firm performance, (b) that procurement practices are necessary (*in kind and degree*) for firm performance, and (c) that these practices are bottlenecks that inhibit firm performance in that they enable positive variations in firm performance [20]. These raises questions as to whether (a) combinations of procurement practices might explain firm performance or (b) certain levels of procurement practices are required to guarantee positive variations in firm performance. To this end, an asymmetric investigation of the procurement practices-firm performance nexus is not only timely but necessary to help uncover the neglected configurational and necessity relationships between procurement practices and firm performance.

Accordingly, this research sought to (a) explore the causal patterns of procurement practices that enhance firm performance and (b) determine the necessity of these practices for firm performance. Specifically, the study ascertains how procurement planning, supplier partnership, contract management, and compliance combine to form sufficient configurations that enhance firm performance and whether any of these practices act as bottlenecks for firm performance. Consequently, the study attempts to answer the following questions:

RQ1 What configurations of procurement planning, contract management, supplier partnership and compliance account for high and low firm performance?

RQ2 (a) Are any, some, or all procurement practices (i.e., procurement planning, contract management, supplier partnership and compliance) necessary for firm performance? and (b) What minimum levels of these practices are required for high levels of firm performance?

To this end, this study endeavours to develop a theory of procurement that clarifies the critical roles of procurement practices in enhancing firm performance. Accordingly, the study mobilised the tenets of complexity theory to deconstruct and test a conceptual model that identifies the asymmetric relationships between procurement practices and firm performance. Consequently, ref. [21] fsQCA (*based on configurational logic*) is used to address research question 1 through the identification of the sufficient complex configurations of procurement practices that lead to high firm performance, and ref. [22] NCA (*based on necessity logic*) for research question 2 via the detection of single necessary but not sufficient procurement practices that allow firm performance [20,23]. Even though fsQCA can determine necessity (in kind), NCA was used to complement fsQCA because this study is not only interested in the procurement practices that are important for a firm's performance (i.e., a *necessity in kind*), but is also interested in figuring out the extent to which these practices are important for a certain desired level of performance (*i.e., necessity in degree*). This is to avoid the situation where a sufficient causal recipe, identified by fsQCA, fails to achieve the outcome because it lacks a minimum degree of a necessary condition [23].

This research makes four significant contributions to theory and practice. First, the study conceptualised procurement as a complex, multifaceted phenomenon comprising four practices that collectively influence firm performance. Second, the study used complexity theory, together with configurational analysis, to put forth a new approach to theory building in procurement management. This was achieved through the identification of complex and asymmetrical recipes of procurement practices for firm performance. Accordingly, procurement has been modelled to include systematically symbiotic rather than independent dimensions with complex combinatorial effects on firm performance. Third, the study complemented fsQCA with NCA by using calibrated set membership scores from fsQCA to perform the NCA analysis. Finally, we integrated the findings from fsQCA and NCA to ensure that the causal recipes have the right levels of the necessary conditions to ensure that they can produce the outcome.

The remainder of this paper proceeds as follows: Section 2 discusses complexity theory as well as existing literature on procurement practices and firm performance. That same section develops and presents the research propositions assessed in the study. Next, section 3 details the chosen research methodology., including the measures, sample and survey instrument, as well as reliability and validity statistics. In section 4, the fsQCA results, including contrarian case analysis, calibration, truth table analysis, necessity, and sufficiency analysis, are presented. Section 5 presents the results of the NCA analysis. The final section includes the discussions, implications, limitations and future research suggestions and conclusions.

## 2 Theory, concepts, and research propositions

### 2.1 Complexity theory

Whereas several theories exist in the literature for explaining the symmetric relationships between causal antecedents and their respective outcomes, complexity theory is deemed one of the most appropriate for deciphering the asymmetric relationships between constructs [24]. Operations and supply chain management studies seek to provide an understanding of the interactions between firms and their supply chain partners through research that captures the complexity of day-to-day practices as they are established and changed over time [25,26]. Complexity, a characteristic of many social phenomena that underpins how outcomes are influenced through the interactions between dynamic systems, processes and practices, has been described as a scientific trend that will dominate the future due to its ability to explain any complex system [27,28]. Complexity theory posits that current symmetric analysis techniques are insufficient to capture and explain situations [29,30].

Fundamental to complexity and configurational theory are the principles of equifinality and causal asymmetry [31,32]. While the tenet of equifinality posits that a complex recipe of causal conditions may lead to an idem consequence which may be explainable by a combination of different causal antecedents, also referred to as sufficient configurations as posited by Ref. [23], the tenet of causal asymmetry, suggests that a recipe that produces an outcome is not a mirror opposite of one that leads to the absence of an idem outcome [33].

Complexity theory has been applied in different fields of business, including supply chain collaboration by Ref. [34], brand equity by Ref. [32], sustainable clothing purchase behaviour by Ref. [24], and mobile gaming by Ref. [35]. Stemming from the systems theory, complexity theory helps explain the non-linear and asymmetric relationships between antecedents and outcomes. This theory suggests that cases understudy can be likened to configurations of interconnected components with complex causal relations characterised with conjunction (outcomes made up of combinations of different causal factors), equifinality (multiple recipes of antecedents lead to an outcome) and asymmetry (factors in a configuration may be immaterial or even contrarywise linked to other recipes) [36].

In recent times, business and management researchers contend that traditional symmetric analysis methods such as regression and SEM, which are underpinned by the assumption of additive, inifinal and symmetrical causality, are not suitable for modelling complex phenomena e.g., Refs. [20,24,25,28,32,33,37]. Accordingly, applying traditional regression-based methods might result in inaccurate results owing to issues of the obliviousness of contrarian cases, multicollinearity, and non-normality [38]. Hence, the asymmetric angle offers business researchers a novel approach to data analysis in their analysis toolbox that enables them to develop and test hypotheses encompassing complex phenomena, thereby enhancing our understanding of business concepts. The premise of this theory is that an outcome is better explained as recipes of symbiotic antecedent constructs. Investigating the combinatorial effects of antecedent constructs permits the exploration of asymmetric links between drivers and outcomes.

In recent times, scholars have used complexity theory along with configurational analysis to assess sophisticated phenomena such as supply chain collaboration by Ref. [34], supplier selection by Ref. [39], impulsive buying behaviour by Ref. [33], foreign direct investment by Ref. [40], green finance development by Ref. [25], business analytics by Ref. [41], Luxury brand purchases by Ref. [42], brand equity by Ref. [32], sustainable clothing purchase behaviour by Ref. [24], service robots by Ref. [28], entrepreneurial ecosystems by Ref. [37].

Along these lines, this study employed complexity theory and a configurational approach to investigate and uncover the complex and asymmetric relationship between procurement practices and firm performance.

### 2.2 Procurement practices

#### 2.2.1 Procurement planning

Procurement is an activity that entails the acquisition of goods, services and works for the satisfaction of a corporate business need or even the various needs of private individuals [43]. Over the past decades, annual reports of corporate organisations have proven that procurement expenses of businesses are responsible for close to 75 % of total organisational expenditure [44]. Thus, because huge sums of financial resources are committed to undertaking procurement activities, reference [45] recommend that due diligence ought to be a requisite skill for procurement professionals when undertaking procurement activities. According to Ref. [46], undertaking due diligence in procurement activities requires effective and efficient procurement process planning before embarking on such an acquisition undertaking. Taking a critical reference from the extant definitions of what constitutes procurement planning, reference [47] advanced that the process of planning procurement to be undertaken is one able to transform the procurement process uncertainties into certainties.

#### 2.2.2 Contract management

Contract management refers to the operational and technical process of ensuring that the contracting obligations of parties to a contractual arrangement are effectively and efficiently executed in a harmonious manner without significant discomfort to any of the parties to the contractual arrangement [48]. These requirements could be in the form of raw materials, consumer goods, machines and equipment, construction in the form of works, rendering of service such as training and consulting [49]. In trying to obtain goods, works or services from trading partners, a business organisation indulges itself in contractual responsibilities and such contracts, whether small, medium or large, needs to be managed effectively and efficiently so as to be able to achieve all business deliverables per the contractual arrangement [50]. The management of performance-related risks, especially other risks, is one of the key reasons why contracts need to be managed by business organisations [51].

#### 2.2.3 Compliance

Compliance with procurement rules and policies regarding social, legal and environmental laws has been observed as a relevant procurement practice with the operational potency to enhance organisational growth or success [52]. It is a well-advanced fact that the activities or operations of business organisations, in one way or the other, negatively affect the sustainability of the triple bottom line [53]. Due to the increasing levels of unsustainable practices among business organisations, institutional activists in charge of ensuring the sustainable preservation of the business and social environments have sought to effectively manage the negative impact of business organisations by stipulating some standards, regulatory codes, as statutory requirements that seek to guide business organisations as to how to undertake their business activities in a more preservative manner [54]. Accordingly, business complying with regulatory, social and environmental standards is seen as a mission to obtain operational legitimacy in the business environment [53].

#### 2.2.4 Supplier partnership

Supplier partnership refers to a contractual, temporary arrangement between independent companies that aims to reduce the ambiguity surrounding the achievement of the partners’ strategic goals by managing or jointly conducting one or more of their activities as posited by Refs. [[55], [56], [57]]. Ref. [58] therefore, advised that supplier relationships incorporate issues such as “co-inventorship, intellectual property ownership, technology transfer, exclusivity, competition, hiring away of employees, rights to business opportunities created during the partnership, splitting of profits and expenses, duration and termination of the relationship”. Laws, economic conditions, and structures, including legal requirements, macroeconomic policies and indices, distribution networks, and contract compliance methods, are only a few of the business considerations that contribute to the need for a strategic partnership [59,60]. Operational performance and competitive advantage can be sought while market uncertainty and hierarchical rigidities are avoided [61]. As new links build on established inter-firm relationships, corporate social capital affects the formation of new partnerships [62].

### 2.3 Firm performance

The performance of business organisations represents a valid indicator of business success or organisational growth [63]. In other words, firm performance can be deemed as that subjective or objective means of assessing whether the business's objectives, goals or targets have been duly met or whether the business organisation has deviated from such predetermined outcomes [64]. According to Ref. [65], the performance of business organisations can be assessed financially and non-financially. The financial performance metrics are a set of performance evaluative indices such as profitability performance, operational performance, share performance, marketing performance etc, determined using financial bases or in financials [66].

Conversely, the non-financial performance metrics represent those forms of performance indicators of business that are not quantifiable in monetary or financial, such as firm reputation and sustainability performance. According to Ref. [67], assessing the performance of a business organisation with both financial and non-financial criteria gives firms snapshots of the true performance that a business organisation is achieving. Reference [68] also affirmed that irrespective of the exact need for a firm performance index, the act of relying on a particular metric of firm performance, such as financial or non-financial, limits the chances of the business to observe the true performance of their organisation. They further stressed that sometimes the outcomes of performances measured in financial terms are seemingly dependent on the state of other non-financial performance metrics which business scientists have not assessed; hence, understanding the practical implications behind a financially skewed evaluated firm performance is vaguely accomplished and this will lead to business organisations implementing biased operational interventions that might not be able to effectively improve firm performance.

### 2.4 Research propositions

#### 2.4.1 Configurational effects of procurement practices on firm performance

Research on the symmetric nexus between procurement practices and firm performance provides the foundation for understanding the relationship between multiple procurement practices and firm performance [17]. In such studies, linear, symmetric, or net effects of procurement practices on firm performance are ascertained, to the neglect of the combinatorial effects of multiple procurement practices on firm performance. A symbiotic and aggressive association exists among the various procurement practices. Hence, firm performance might be influenced by how procurement practices are linked, combined, and matched. Therefore, a configurational approach is needed to ascertain how the multiple procurement practices can be configured to explore necessity as well as sufficient causal recipes that enhance firm performance.

The configurational perspective through complexity theory clarifies how conditions combine to explain an outcome. It suggests that organisations are collections of interrelated units and practices instead of isolated units or loosely combined entities. The notion that firms cannot comprehend complexity through analysing their isolated components or practices is consistent with our understanding of procurement and its practices. Due to the nonlinearity, equivalence, and asymmetry of the causal relationships between procurement practices and firm performance, the configurational viewpoint is ideally suited for analysing these relationships. Since statistical hypotheses and matrices are not appropriate for investigating complex phenomena, we used propositions to represent the combinations of testable precepts associated with the identification of complex procurement practices that enhance performance [26]. Complexity theory suggests that combinations of the same group of conditions can produce different outcomes by enhancing or diminishing an outcome [35]; hence, this study assesses the combinations of procurement planning, supplier partnership, contract management, and compliance that predict firm performance.

The fundamental tenet of complexity theory, equifinality, proposes that an outcome (endogenous construct) can be explained by different combinations of causal conditions that combine to produce sufficient causal recipes. Put differently, the same outcome can be achieved from more than one combination of causal antecedents [26]. In this study, procurement planning, supplier partnership, and contract management and compliance are key causal conditions that may be configured into different configurations to enhance firm performance. For instance, Ref. [17] suggest that firm performance is associated with procurement practices such as supplier partnership and effective contract management. Also, reference [56] also found that procurement planning and compliance are key determinants of firm performance. Hence, configurations may include combinations of procurement planning, supplier partnership, contract management and compliance, resulting in the following propositions. Based on the principle of equifinality, it is proposed that.

Proposition 1a *There is no optimal combination of procurement practices (i.e., procurement planning, contract management, supplier partnership, and compliance) that is sufficient for high firm performance; rather, there exist multiple pathways through which these practices enhance firm performance. (Principle of equifinality)*

The second principle of complexity theory, the principle of causal asymmetry, does not support the logic behind the net-effect theory, which assumes linear or symmetric relationships between causes and their outcomes [26]. Instead, it supposes asymmetric relationships between conditions and their outcomes such that a causal condition assumes different and even contrasting effects on an outcome of interest, but contingent on how the said causal condition fuses with the other causal factors used [25]. For instance, because procurement contribution to firm performance has been varied, it can enhance firm performance either through high levels of procurement planning, but this will depend on how procurement planning combines with supplier partnership, contract management and compliance. On the basis of the causal asymmetry principle, it is suggested that.

Proposition 1b *Single procurement practices (i.e., procurement planning, contract management, supplier partnership, and compliance) can have opposite effects on firm performance, but this is contingent on how they combine with other procurement practices, though no single combination of these practices is a mirror opposite (Principle of Causal asymmetry)*

#### 2.4.2 Procurement practices as necessary conditions for firm performance

Reference [69] opined that firms that take stringent approaches to planning their procurement process can aid the acquisition process with respect to being able to collect similar procurement requirements, dividing complex procurement requirements into lots and even prioritising the business needs so as not to mount excessive pressures/stress on the business’ finances. Hence, procurement planning acts as the catalyst for any successful procurement endeavour.

Furthermore, due to the capital requirement of some technical or complex contracts to be executed, contract management is deemed prudent as it will help to control cost, introduce contractual change in a more logical and objective manner, manage and maintain a cordial relationship with trading partners with whom the business is contracted [70]. Hence, all activities that transpire as the contract is being executed need to be well monitored and assessed, and where deviations are observable, desirable remedies are put in motion to correct such undesirable inconsistencies [71]. Moreover, the success of a contractual undertaking depends on how the business organisation develops or chooses an appropriate approach for monitoring the key performance indicators (KPIs) of an ongoing contract [72].

Gaining legitimacy in the business environment connotes having the legal right to operate as a business entity, enjoying increased and continuous patronage from trade partners, easy access to resources (human and financial), and a peaceful serenity to operate the business without being distracted by legal suits, legal fines, operational halts, business close-downs etc. [73]. Hence, when business organisations fail to comply with agreements and operational standards within the business environment, their naivety or ignorance of such compliance practices causes huge losses to such business organisations and some applicable sanctions such as seized operational license, huge legal fines, unpatronised products, or services by customers to the business, and high employee turnover [74].

Strategic partnerships contribute significantly to the sustainability and competitiveness of the company's operations [56]. Improved business performance is a result of information exchange, growth in market share, enhanced quality, inventory reductions, a quicker product development cycle, and better delivery services [75,76]. The ability of companies to share their core competencies, specialisations, and expertise learned over time is a vital component of information sharing [77]. The strategic partnership also increases operational performance by enhancing logistics and distribution networks, increasing market share.

Prior studies suggest that procurement affects firm performance by affecting operational performance and financial performance [78]. Procurement planning prevents maverick buying, thereby minimising the costs of bought-out items. Supplier partnerships endanger trust and minimise supplier opportunistic behaviour. However, these studies have failed to indicate whether these procurement practices are necessary for firm performance besides identifying the levels of these practices that guarantee firm performance. Consequently, if the necessary levels of procurement planning, supplier partnership, contract management or compliance are not secured, firm performance will fall. Further, if a necessary procurement practice is absent from a causal configuration, it cannot be guaranteed that the causal configuration will lead to firm performance. Hence, for firms to guarantee enhanced performance levels, they must (a) provide a necessary procurement practice and (b) at a minimum level. Therefore, it is proposed that.

Proposition 2 *Procurement practices, in the form of procurement planning*, *supplier partnership*, *contract management and compliance*, *are necessary (“but not sufficient”) for firm performance.*

### 2.5 Research framework

Fig. 2 shows the research framework for this study. Having carefully scrutinised the literature on procurement, four commonly used procurement practices were identified as phenomena that enhance or diminish firm performance. Accordingly, the configurational model (Model A) in Fig. 2 portrays procurement planning, supplier partnership, contract management and compliance as causal conditions that combine to enhance or diminish firm performance. Similarly, the conceptual model (Model B) in Fig. 2, procurement planning, supplier partnership, contract management and compliance, were modelled as necessary conditions for firm performance.

## 3 Method

### 3.1 Sample

Procurement professionals from private universities in Ghana were sampled. This study sought to ascertain how implementing procurement practices enhances firm performance through profitability, the study used only private universities as public universities were not set up to make profits. Before data collection, the researcher presented a letter introducing the study and the researchers signed by the Head, Department of Marketing and Supply Chain Management of the University of Cape Coast to all the institutions and the respondents involved in the study. After that, and before beginning the survey, each respondent was asked to read and sign an informed consent form. From a population of 312 procurement professionals from 78 private universities, a sample of 220 respondents was given the questionnaires, out of which 156 were retrieved, and 150 were useable. The useable sample consisted of about 60.3 % males and 39.3 % females. A bachelor's degree was held by the majority of respondents. (62 %), while about 36.7 % had postgraduate degrees. Additionally, the majority of respondents (52 %) are in the age range of 31–40 years, while 18.7 % and 15.3 %, respectively, are in the 20–30 year old and over 50-year-old age brackets.

### 3.2 Survey instrument

The survey instrument consists of two parts; whereas section A consisted of questions on the sample's demographics, section B was made up of questions (identified in the literature review) that measure the various constructs in the study. Regarding Procurement Planning, the works of reference [79], reference [18], reference [80], and reference [81] were adapted. Exemplar items include “Planning for procuring consultancy services”, “Planning for procuring technical services”, “Taking inputs from external stakeholders”, “Using inputs from the annual estimated budget for procurement planning”. For the contract management construct, the works of reference [82], reference [83], and reference [48] were adapted. Sample items include “Achieving required quality service level”, “Achieving low supplier defect rates”, “Communicating changes in contracts to all contracts”, and “Maintaining a good relationship with contracting parties”. In measuring the Supplier Partnership construct, items such as “Monitoring materials and supplier”, and “Suppliers are included in continuous improvement programs”, adapted from the works of reference [84], reference [85], and reference [86] were used. Finally, the organisational Performance construct was measured with items such as “Improved value for money”, “Promoting efficiency in procurement procedure”, “Eliminating contracts breaches”, and “Enhancing timely response to customer needs” adapted from works of reference [87], reference [88], reference [89], and reference [90]. All constructs were measured with a seven-point Likert scale, anchored from 1 to 7, where 1 and 7 denote (“strongly disagree”) and (“strongly agree”) respectively.

### 3.3 Reliability and validity

First, the validity and reliability of the study's conditions and outcomes were evaluated. Cronbach's alpha values varied from 0.764 to 0.926, while the composite reliability values were higher above the 0.7 cutoff point and ranged from 0.864 to 0.937. To assess convergent validity, the average variance extracted was used. The AVEs for the constructs exceeded the cut-off threshold of 0.5 and ranged from 0.579 to 0.680. Finally, discriminant validity was assessed with the HTMT ratio, where all HTMT values were below 0.850 and ranged from 0.611 to 0.818, and all the biased corrected confidence interval values were less than 1. Appendix A presents results that confirm the reliability and validity of the constructs (conditions and outcomes) used in the study.

## 4 fsQCA

### 4.1 Contrarian case analysis

**Fig. 1:**
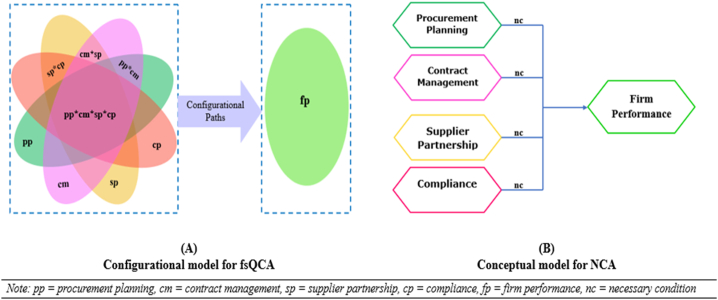
Research framework.

To determine whether inverse relationships occur in the data set, thereby necessitating the deployment of configurational analysis, contrarian analysis was performed as suggested by Refs. [29,91], and [92]. The results (Fig. 1) show the occurrence of (1) low to very low levels of compliance leading high to very high levels of firm performance (3 + 0+4 + 2 = 9 cases or 9/150 or 6 % of the total cases) along with low to very low levels of compliance resulting in high to very high levels of firm performance (1 + 1+0 + 0 = 2 or 2/150 or 1.3 % of total cases), (2) low to very low levels of contract management leading high to very high levels of firm performance (0 + 0+10 + 2 = 12 cases or 12/150 or 8 % of the total cases) together with low to very low levels of compliance resulting in high to very high levels of firm performance (2 + 5+1 + 8 = 16 or 16/150 or 10.7 % of total cases), (3) low to very low levels of procurement planning leading high to very high levels of firm performance (0 + 1+13 + 5 = 19 cases or 19/150 or 12.7 % of the total cases) in addition to low to very low levels of procurement planning resulting in high to very high levels of firm performance (3 + 1+0 + 8 = 12 or 12/150 or 8 % of total cases) and (4) low to very low levels of supplier partnership leading high to very high levels of firm performance (6 + 0+10 + 1 = 17 cases or 17/150 or 11.3 % of the total cases) in addition to low to very low levels of supplier partnership resulting in high to very high levels of firm performance (0 + 13+3 + 2 = 18 or 18/150 or 12 % of total cases).

**Fig. 2:**
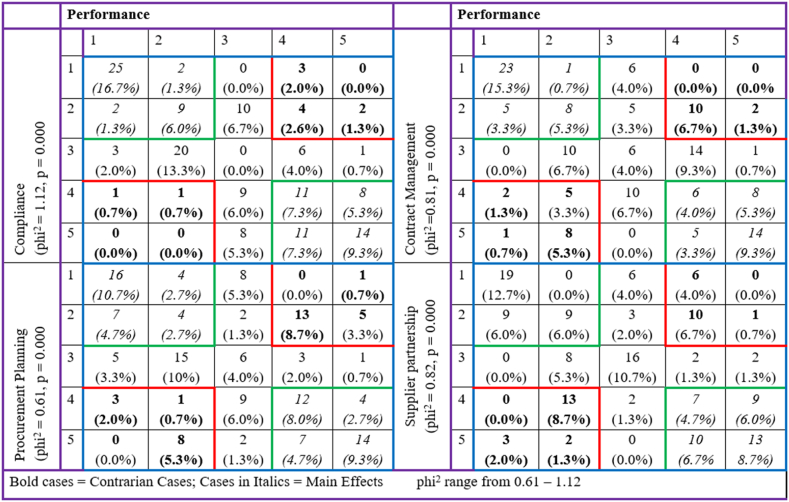
Contrarian case results.

Accordingly, more than 7.3 % (for the compliance and firm performance nexus), 18.7 % (for the contract management and firm performance nexus), 20.6 % (for the procurement planning and firm performance nexus), and 23.3 % (for the supplier partnership and firm performance nexus) of the cases exhibit two relationships that counteract the relationships that suggest that high levels of procurement planning, supplier partnership, contract management and compliance increase firm performance. With these contrarian cases, a symmetric analysis is not sufficient in explaining the relationships existing in the data set, thereby supporting the deployment of asymmetric (configurational) analysis to help explain the asymmetric relationships between the procurement practices on hand and firm performance on the other hand [29,91,92].

### 4.2 Calibration

Having shown that the data set contains contrarian cases, confirming the need for configurational analysis, the study proceeded to calibrate the data into fuzzy sets. The direct method was used to calibrate the data where 95 %, 50 % and 5 % were chosen as threshold values that represent “fully-in”, “cross-over” and “fully out” values, respectively. The resultant values, after applying these percentiles to the data set, together with other descriptive statistics of the data, can be found in Table 1.

**Table 1:** Calibration and descriptive statistics.

Variable	Fuzzy set calibration			Descriptive statistics				
	Fully-out	Cross-over	Fully-in	Mean	Std. Dev.	Min	Max	N
Procurement Planning	4.86	5.46	6.04	5.433	0.513	3.917	6.917	150
Contract Management	4.57	5.57	6.01	5.461	0.555	4.214	6.786	150
Supplier Partnership	4.95	5.50	6.00	5.502	0.548	4.000	7.000	150
Compliance	4.75	5.50	6.00	5.513	0.650	3.250	7.000	150
Performance	5.00	5.75	6.33	5.666	0.533	4.000	7.000	150

### 4.3 Truth table analysis

The next logical step after calibrating the data into fuzzy sets is to create the truth table that includes 2 k rows “where k refers to the number of independent constructs and the rows denoting the likely combinations of the independent constructs” [93] by performing truth table analysis. In creating the truth table (Appendix B), a consistency threshold of 80 % was used, whereas all rows with less than two cases were removed. Hence, the truth table includes all possible combinations of procurement practices (i.e., procurement planning, contract management, supplier partnership, and compliance. Appendix B suggests that the only way a >0.91 consistency can be achieved is when all the procurement practices being investigated are present. Investigating the consistency and coverage of all configurations, including various numbers of procurement practices, is crucial, nevertheless [93].

### 4.4 Necessity analysis

Having performed the truth table analysis and created the truth table (Appendix B), necessary conditions were tested for the presence or absence of firm performance and the results are presented in Table 2. More specifically, the consistency values for firm performance ranged from 0.745 to 0.895 and 0.519 to 0.735 for ‘high’ firm performance and ‘not high’ firm performance, respectively. Since none of the causal conditions had consistency values ≥ 0.9, individually, each is considered necessary but not sufficient for high levels of firm performance. Accordingly, the analysis proceeded with sufficiency analysis to identify sufficient configurations of procurement practices that account for high firm performance. This is presented in the next section.

**Table 2:** Analysis of necessity.

	High firm performance		Low firm performance	
	Consistency	Coverage	Consistency	Coverage
Procurement Planning	0.745	0.707	0.519	0.556
∼Procurement Planning	0.532	0.494	0.726	0.763
Contract Management	0.815	0.731	0.544	0.551
∼Contract Management	0.500	0.492	0.735	0.818
Supplier Partnership	0.824	0.717	0.539	0.529
∼Supplier Partnership	0.459	0.468	0.711	0.820
Compliance	0.895	0.728	0.575	0.528
∼Compliance	0.420	0.466	0.704	0.883

### 4.5 Sufficiency analysis

After the necessity analysis, the study proceeded to determine the combinations of procurement planning, supplier partnership, contract management and compliance that are sufficient for high firm performance. The results of fsQCA analysis on the recipes of procurement practices with acceptable consistency (>80 %) and coverage (>20 %) accounting for high firm performance as well as medium to low firm performance are presented in Table 3. In this, conditions may either be present, absent, or negated. In Table 3, all possible configurations for high as well as low levels of firm performance are presented, with their accompanying consistency (>0.75) and coverage values, respectively. Whereas consistency denotes the degree to which these combinations of causal conditions are a part of the solution, coverage denotes the empirical relevance of the outcome [94]. Table 3 presents the combinations of procurement practices that are sufficient for both enhanced and diminished levels of firm performance. Whereas three (M1, M2 & M3) complex configurations (coverage = 0.873, consistency = 0.756) emerged for the presence of the outcome (firm performance), four recipes (M4, M5, M6 & M7) of procurement practices (coverage = 0.808, consistency = 0.819) were obtained for its absence. Altogether, the consistency values exceeded the recommended threshold value (>0.75). For the presence (absence) of firm performance, the overall solution coverage of 83.7 % (80.8 %) suggests that the three (four) recipes explain a substantial proportion of high (low) performance among the firms.

**Table 3:** Analysis of sufficiency.

	Models for high firm performance				Models for not high firm performance				
		**1**	**2**	**3**		**4**	**5**	**6**	**7**
Procurement Planning									
Contract Management									
Supplier Partnership									
Compliance									
Row Coverage		0.430	0.750	0.584		0.619	0.406	0.324	0.269
Unique Coverage		0.090	0.085	0.033		0.271	0.028	0.039	0.059
Consistency		0.756	0.830	0.838		0.913	0.806	0.832	0.851
*Overall Solution coverage:*	0.873				0.808				
*Overall Solution consistency:*	0.756				0.819				

Note: “Large, full black circles = presence of core condition; large, crossed open circles = absence of core condition; small, full black circles = presence of peripheral condition; small, crossed open circles = absence of peripheral condition”.

Table 3 also presents the empirical relevance of each causal configuration in the form of raw and unique coverage. While the raw coverage denotes proportion of the outcome that is explained by a specific causal recipe, the unique coverage explains the amount of the outcome that is exclusively accounted for by a specific solution. Accordingly, the recipes presented in Table 3 explain firm performance at varied degrees, ranging from 2.8 % to 27 %. The models for high levels of firm performance, models 1–3 present recipes in which at least one procurement practice is present (i.e., high), and models 4–7 present recipes in which at least one of the procurement practices is absent (i.e., low).

### 4.6 Analysis of configurational paths driving firm performance

Specifically, firm performance increases with high levels of compliance in combination with absence of supplier partnership regardless of the levels of contract management and procurement planning (Model 1). This highlights the importance of compliance with procurement rules and procedures in achieving organisational objectives. It also confirms the role of compliance in contract management because when rules and procedures contained in procurement contracts are complied with, then contract management becomes easy. This model explains 43 % of the variation in firm performance. According to Model 2, 75 % of the cases suggest that high levels of firm performance is guaranteed when there are high levels of both contract management and compliance, regardless of procurement planning and supplier partnership. This conclusion is supported by a solution consistency of 75.6 %. This finding indicates that procurement practices in the form of contract management and compliance are sufficient for achieving high firm performance and underscores the importance of contract management and compliance with rules and regulations for business success. This model explains the largest part of the cases responsible for high levels of firm performance.

In line with Model 3, 58.4 % of the cases suggest that high levels of firm performance are assured with high levels of procurement planning, contract management and supplier partnership. Like the first two solutions, this finding is also backed by a solution consistency of 75.6 %. Remarkably however, compliance was not relevant to this solution, suggesting that high levels of contract management, procurement planning, and supplier partnership are sufficient to produce high levels of firm performance. It is interesting to note that, each of compliance and contract management are important and present in at least two out of the three solutions responsible for high firm performance. In at least two solutions that led to high performance (Models 1 and 2) compliance is present and is combined with at least one other procurement practice (factor) to explain firm performance, thereby confirming the asymmetric relationships.

### 4.7 Analysis of configurational paths not driving firm performance

Models 4, 5, 6 and 7 (Table 3) present causal recipes of procurement practices that are responsible for low levels of firm performance. As suggested by model 4, absence of both contract management and supplier partnership, regardless of the levels of procurement planning and compliance will result in low levels of firm performance. This finding is not surprising because not complying with rules would lead to several problems both within and without the organisation that pose serious threats to firm performance. The model explains 61.9 % of the cases that lead to low levels of firm performance.

For Model 5, absence of supplier partnership and high levels of compliance are responsible for absence of firm performance. This is an interesting finding, considering the importance of high levels of compliance to firm performance. However, this happens when there is absence of supplier partnership, regardless of other procurement practices such as contract management and procurement planning, with the model explaining and accountable for 40.6 % of the cases. Model 6 suggests that 32.4 % of the cases contend that absence of procurement planning, contract management and compliance is sufficient for low levels of firm performance. This model has a consistency of 81.9 %. Also, Model 7 proposes that 26.9 % of the cases propose that low levels of firm performance exist amongst firms with high levels of procurement planning, contract management and supplier partnership in combination with absence of compliance. Accordingly, compliance pays a key role in this model; a low level of compliance results in low levels of firm performance even though all other practices are at a high level.

### 4.8 Predictive validity

**Fig. 3:**
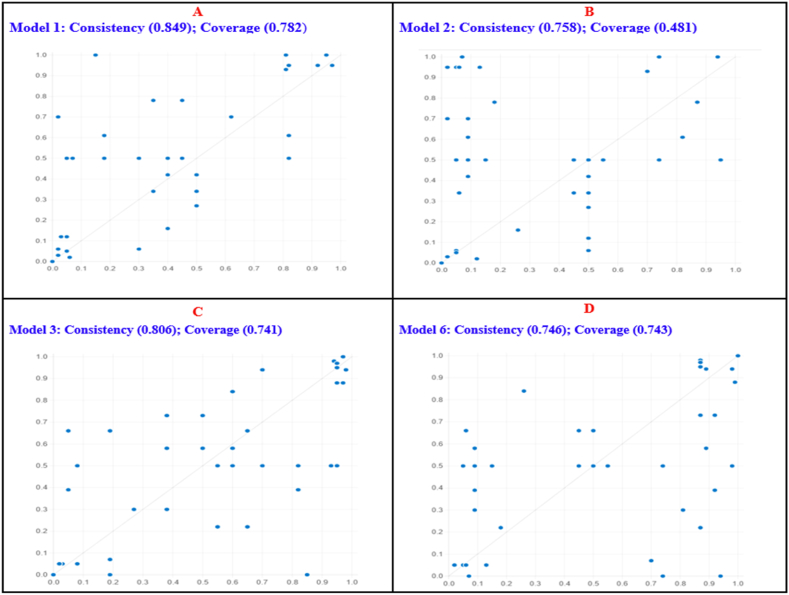
Plots for predictive validity.

To assess if the model can adequately predict the same endogenous construct on different samples of data, predictive validity was examined [29]. Accordingly, the sample was randomly divided into two, sub-sample 1 and sub-sample 2, and fsQCA analysis was performed on sub-sample 1 (see results in Appendix C). The results from sub-sample 1 (Appendix C) were then assessed against those for sub-sample 2 (Fig. 3). Panels A, B, C, and D (Fig. 3) represent plots of the fsQCA results for Models 1,2,3 and 4 (Appendix C) against results from sub-sample 2. It is observed from Fig. 3 that the patterns of complex driver conditions are consistent indicators for high and low levels of firm performance for sub-sample 1, with consistency and coverage of 0.792 (0.747) and 0.763 (0.854) for high (low) firm performance, respectively.

## 5 NCA results

### 5.1 Necessary condition analysis

The object of conducting the NCA analysis was to ascertain the degree of necessity of procurement planning, contract management, supplier partnership and compliance for firm performance. Hence, after performing the fsQCA analysis to determine sufficient configurations of procurement practices for firm performance, the study proceeded by using the NCA package for R v3.0.3 as proposed by Ref. [22] to conduct the NCA analysis. This produced parameters and the bottleneck table for the two distinct ceiling lines – the CE-FDH and the CR-FDH.

**Fig. 4:**
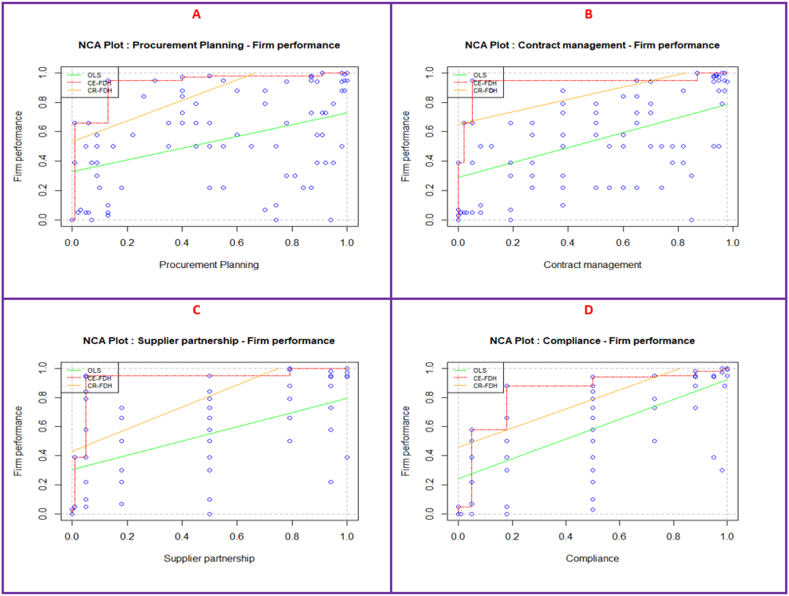
NCA scatterplots.

Fig. 4 shows the scatter plots for procurement practices and firm performance. Panel A portrays the nexus between procurement planning and the performance of a firm, whereas Panel B visually demonstrates the relationship between contract management and firm performance. Similarly, Panel C symbolizes the link between supplier partnership and firm performance, and lastly, Panel D signifies the relationship between compliance and firm performance. As can be observed, each of these plots contain empty spaces in the upper left corner, implying the presence of necessary condition between firm performance (the outcome) and procurement practices (the conditions). Also displayed in these plots are three different ceiling lines: (1) “ordinary least squares (OLS)”, (2) “ceiling envelopment with free disposal hull (CE-FDH)” and (3) ceiling regression with free disposal hull (CR-FDH). Whereas both lines use the observation that are close to the ceiling zone, the CE-FDH and CR-FDH uses “a pairwise linear line” and “a continuous linear line” respectively, [95]. For this analysis, the CR-FDH line is used because it improves on the CE-FDH line and is less sensitive to outliers and measurement error besides being most appropriate for survey data [[95], [96], [97], [98]].

This study presents several criteria for assessing NCA results of which we discuss the effect size and condition inefficiency. For this study, whereas the effect size values (Table 3) ranged from 0.037 (supplier partnership) to 0.172 (compliance), conditional inefficiency values ranged from 50.00 % (supplier partnership) to 94.00 % (procurement planning), meaning that supplier partnership level of above (100–50 = 50.00) is not necessary even for the maximum levels of firm performance. Likewise, condition insufficiency levels for procurement planning and compliance were 94.00 % and 18.00 % (Table 3) respectively suggesting that procurement planning and compliance levels above 6 % and 82 % (Table 3) respectively are not required for even the highest level of firm performance.

The bottleneck table (Table 5) indicates the threshold values for procurement planning, contract management, supplier partnership and compliance are independently necessary for realising the desired level of firm performance. In determining the critical values for firm performance “0 %–30 %, 31 %–70 %, and 71 %–100 %” were used for “low, medium, and high levels” respectively [98]. Hence, procurement planning (5.0), contract management (6.0) and compliance (5.0 %) are the only procurement practices required for achieving low levels of firm performance. Nevertheless, apart from supplier partnership, the remaining three procurement practices (procurement planning, contract management, and compliance) are necessary for medium degrees of firm performance. However, for enhanced firm performance, all four procurement practices were required albeit at varied degrees of necessity. Even so, for the highest level of firm performance (i.e., at 100 %), compliance (82 %) is most necessary, afterwards, supplier partnership (50.0 %) contract management (13.3 %), and procurement planning (6.0 %). The effect size for all conditions and outcomes were examined for their significance. The results (Table 4) suggest that apart from procurement planning and supplier planning, which had large (d > 0.3) and small (d > 0) effect sizes, respectively, contract management and compliance had moderate effect sizes (0 < d < 0.2).

**Table 4:** Results of necessary condition analysis.

Construct	Method	Accuracy	Ceiling Zone	Scope	Effect size (d)	P value	Condition Inefficiency	Outcome Inefficiency
Procurement Planning	CE - FDH	100	0.037	1.000	0.037	0.004	94.000	05.000
	CR - FDH	97.3	0.031	1.000	0.031	0.029	93.349	06.045
Contract Management	CE - FDH	100	0.076	0.980	0.078	0.000	86.735	06.000
	CR - FDH	96	0.056	0.980	0.057	0.021	87.173	10.850
Supplier Partnership	CE - FDH	100	0.120	1.000	0.120	0.000	50.000	61.000
	CR - FDH	97.3	0.080	1.000	0.080	0.000	51.224	67.061
Compliance	CE - FDH	100	0.172	1.000	0.172	0.000	18.000	5.000
	CR - FDH	96	0.247	1.000	0.247	0.000	29.410	30.042

**Note(s) “**CE-FDH = ceiling envelopment with free disposal hull; CR-FDH = ceiling regression with free disposal hull”.

**Table 5:** Bottleneck Levels (in percentages).

Y FP ( %)	X_1_ Procurement Planning		X_2_ Contract Management:		X_3_ Supplier Partnership:		X_4_ Compliance:	
	CE-FDH NC from 5.0 %	CR-FDH NC from 6.0 %	CE-FDH NC from 6.0 %	CR-FDH NC from 10.9 %	CE-FDH NC from 61.0 %	CR-FDH NC from 67.1 %	CE-FDH NC from 5.0 %	CR-FDH NC from 30.0 %
0	NN	NN	NN	NN	NN	NN	NN	NN
10	1.0	0.3	1.0	NN	NN	NN	1.0	NN
20	2.0	1.0	2.0	1.3	NN	NN	2.0	NN
30	2.0	1.7	3.1	2.8	NN	NN	2.0	NN
40	2.0	2.4	3.1	4.2	NN	NN	2.0	10.0
50	2.0	3.1	3.1	5.6	NN	NN	2.0	20.1
60	5.0	3.8	13.3	7.1	NN	NN	2.0	30.2
70	6.0	4.5	13.3	8.5	6.0	4.4	2.0	40.3
80	6.0	5.2	13.3	9.9	50.0	19.2	50.0	50.4
90	6.0	5.9	13.3	11.4	50.0	34.0	50.0	60.5
100	6.0	6.7	13.3	12.8 6	50.0	48.8	82.0	70.6

**Note(s) “**FP = firm performance; CE-FDH = ceiling envelopment with free disposal hull; CR-FDH = ceiling regression with free disposal hull; NN = not necessary”.

### 5.2 Complementing fsQCA with NCA

To complement the fsQCA finding with NCA, NCA approach by Ref. [23] was performed with the calibrated scores (set membership scores). According to Refs. [21,22], a condition is necessary when the effect size (d) is ≥ 0.10 and the significance is < 0.05. Table 4 shows that the effect sizes (p values) for procurement planning, contract management, supplier partnership and compliance were 0.037 (0.004), 0.078 (0.000), 0.120 (0.000), and 0.172 (0.000), respectively. Since supplier partnership and compliance met reference [21] criteria of having an effect size of at least 0.10 and a p-value of less than 0.05, they are deemed to be necessary for firm performance [99,100].

However, the results (Table 4) show that procurement planning and contract management failed to pass the two-condition test of necessity ref. [21,100] because, even though both constructs had p values less than 0.05 (i.e., 0.004 and 0.000 for procurement planning and contract management respectively), their effect sizes (d) were small (i.e., 0.037 and 0.078 for procurement planning and contract management respectively). Hence, even though these two constructs (procurement planning and contract management) are statistically significant, they are not practically significant, hence, are deemed not to be necessary for firm performance [21,100].

### 5.3 Integrating fsQCA and NCA findings

The NCA findings (Table 4) indicate that the presence of supplier partnership >0.12 as well as compliance >0.17 are necessary for firm performance above >0.5. These requirements have been achieved in recipes 1 and 3 for supplier partnership and recipes 1 and 2 for compliance. However, same cannot be said of recipe 2 for supplier partnership and recipe 3 for compliance. Moreover, these are necessary requirements, hence without them, the configurations cannot lead to firm performance. However, none of the recipes identified by fsQCA includes all necessary conditions (i.e., procurement planning, contract management, supplier partnership and compliance) as identified by NCA. Accordingly, the requirement of supplier partnership >0.12 is added to recipe 2, and compliance >0.17 is added to recipe 3 to ensure that these recipes can produce the outcome. These combinations are presented in Table 6.

**Table 6:** Analysis of sufficiency with required necessity requirements.

		Sufficient configurations			Necessity Requirement
		1	2	3	
Procurement Planning					
Contract Management					
Supplier Partnership					>12.0 % (0.12)
Compliance					>17.2 % (0.17)
Row Coverage		0.430	0.750	0.584	
Unique Coverage		0.090	0.085	0.033	
Consistency		0.756	0.830	0.838	
*Overall Solution coverage:*	0.873				
*Overall Solution consistency:*	0.756				

Note: Large, full black circles = presence of core condition according to QCA; large, crossed open circles = absence of core condition according to QCA; small, full black circles = presence of peripheral condition according to QCA; small, crossed open circles = absence of peripheral condition according to QCA; full black squares = minimum required necessity membership according to NCA.

## 6 Discussion of the results

Prior scholars have explored the link between procurement practices and firm performance [13,14]. However, understanding how procurement accounts for the complexity of variations in firm performance has remained unexplored, thus creating a gap in the procurement literature that calls for attention. To this end, this study proposes a configurational model of procurement practices as symbiotic conditions to explore the combinations of these practices that lead to high and low firm performance. Accordingly, the study sought to achieve two main objectives. First, we modelled procurement practices (procurement planning, contract management, supplier partnership, and compliance) as complex causal configurations for firm performance. Second, we examined the necessity of these procurement practices (procurement planning, contract management, supplier partnership, and compliance) for firm performance. Accordingly, the model was examined in two distinct, but complementary ways. Firstly, a fuzzy set qualitative comparative analysis was performed to determine sufficient causal recipes of procurement practices (i.e., procurement planning, contract management, supplier partnership, and compliance) for firm performance. Secondly, necessary condition analysis was performed to investigate the degrees of the necessity of procurement planning, contract management, supplier partnership, and compliance relevance for firm performance.

Findings from fsQCA suggest that, whereas three sufficient complex causal recipes of procurement practices lead to high levels of firm performance, four distinct, but sufficient causal configurations of procurement practices lead to low levels of firm performance. Hence an assessment of the findings reveals that, though none of the procurement practices was necessary for low levels of firm performance, their existence is essential for moderate and high levels of firm performance. Particularly, firms with low firm performance who desire a higher level of firm performance should first enhance procurement by making the appropriate investment into their procurement planning, contract management, supplier partnership, and compliance activities. Likewise, firms with moderate levels of firm performance who desire to upgrade their performance to higher levels must improve their procurement planning, contract management, supplier partnership, and compliance. Our results also indicate that very high levels of firm performance are achievable when firms invest in and improve upon their contract management, supplier partnership, and compliance with procurement rules and policies.

The study employed NCA in conjunction with fsQCA, following recommendations of reference [23], and [96] who suggested that NCA should be used together with fsQCA for a more nuanced understanding of necessity. The NCA findings highlight that all conditions (procurement planning, contract, management, supplier partnership, and compliance) display different degrees of necessity for the outcome (firm performance). The NCA results further indicate that the largest effect size on firm performance was for procurement planning (d = 0.372), with contract management (d = 0.243), compliance (d = 0.237), and supplier partnership (d = 0.1,69) following in that order. Also, the findings for conditional inefficiency suggest that for very high levels of firm performance (>70 %), 66.62 %, 36.32 %, 31.5 %, and 19.62 %, are required as maximum levels of procurement planning, contract management, supplier partnership, and compliance respectively. These findings enlighten procurement and supply chain managers to pay attention to the necessary levels of these practices. Besides, improving the levels of these practices over and above the required levels does not result in any differential increase in firm performance. Finally, whereas necessity analysis using fsQCA's suggests not a single procurement practice is necessary for firm performance, the necessity analysis via NCA reveals that substantial investment and improving in all the procurement practices is required to attain higher levels of firm performance.

### 6.1 Theoretical and methodological implications

The implications of this study are six-fold. First, the study examined procurement practices as drivers of firm performance. In the digital procurement and supply chain literature, supply chain management practices such as (with one or two procurement practices) are widely modelled as predictors of firm performance. However, the effect of procurement practices (though very crucial to savings and value for money) on firm performance has been widely ignored in the literature. Accordingly, this study modelled procurement planning, contract management, supplier partnership, and compliance as drivers of firm performance. Hence this study may be the first to specifically identify and assess the roles of procurement practices in driving firm performance, thereby expanding our understanding of the roles of procurement practices in firm performance improvements.

Second, this study, via a configurational approach, developed and assessed a model that explored how key procurement practices combine to predict firm performance. The study conceptualised procurement as a complex and multidimensional construct made up of four independent dimensions and proposed a configurational recipe of these dimensions that drives firm performance. The combinatorial/asymmetrical perspective employed in this study constitutes a worthwhile addition to prior research (which was mainly symmetrical) on the drivers of firm performance. This new perspective differentiates this study from prior studies by enabling the assessment of asymmetric relationships between procurement practices and firm performance besides enabling the identification of combinatorial recipes of procurement practices that drive both high and low firm performance.

Third, the fsQCA results highlight that different procurement practices had varied degrees of importance besides each practice interacting and combining with other practices thereby enhancing our understanding of how procurement contributes to firm performance. However, the NCA results indicate that procurement planning had the largest effect size, highlighting the significance of planning in procurement. This finding adds to our appreciation of how procurement drives firm performance.

Fourth, prior studies used sufficiency logic (i.e., either SEM or multiple regression) as a single method to investigate the role of procurement in enhancing firm performance, with few researchers using either fsQCA, NCA, or both. However, whereas QCA is based on fuzzy. logic, and hence is capable of effectively examining different causal combinations of drivers, by contrast, NCA is built on the necessary logic, where one construct cannot offset the effect of the another. Accordingly, we combined fsQCA and NCA to provide a more nuanced understanding of the mechanisms through which procurement practices improve firm performance. Accordingly, the NCA results underscore the importance of the “must-have” antecedent constructs in enhancing the outcome construct. Therefore, procurement practices are “necessary but not sufficient” for firm performance. Hence the presence of these practices is necessary but does not guarantee firm performance, even though the absence of these practices does not guarantee failure either.

Fifth, this study proves that a combination of procurement planning, contract management, supplier partnership, and compliance drive high levels of firm performance thereby shedding light on the importance of procurement for firm performance. It therefore offers a more nuanced understanding of the different configurations of procurement practices that lead to either high or low firm performance.

Sixth, this research constitutes one of the foremost scholarly endeavours in supply chain management that complements fsQCA with NCA in assessing the complex relationships between antecedents and outcomes. Accordingly, whereas fsQCA permitted the examination of the asymmetric relationship between procurement practices and firm performance, NCA not only facilitated the identification of the procurement practices that are required for enhanced firm performance but also supported the determination of the levels of these procurement practices that are necessary for producing specific levels of firm performance. In particular, the paper demonstrates that distinct degrees of firm performance necessitate distinct levels of individual procurement practices.

### 6.2 Managerial implications

This paper proffers new perspectives and ways to achieve high levels of firm performance through diverse configurations of procurement practices. Operationalisation of these recipes may lead firms to the desired levels of firm performance; thus, supply chain managers should use these proposed causal configurations to guide the development, design, and investments in procurement practices and policies to ensure the achievement of high levels of firm performance. Hence this study offers a five-fold implication for procurement and supply chain practitioners.

First, it offers insight to procurement and supply chain managers on how to streamline investments in procurement practices (i.e., procurement planning, contract management, supplier partnership, and compliance) by identifying specific causal recipes that can lead to high firm performance. The identified configurations provide new ways on how firms may approach procurement activities as each recipe covers specific aspects of the sample. Thereby, underscoring the fact that there is no single causal recipe of procurement practices that explains firm performance. Procurement practitioners may invest in procurement practices that help improve procurement efficiencies to achieve value for money. Since firm performance is based on the efficient and effective use of a firm's resources, procurement provides more insight into how a firm can improve its performance. Accordingly, procurement practices allow procurement practitioners to plan and anticipate inherent risk in their interactions with their external partners (i.e., suppliers).

Second, the NCA findings highlight the significance of procurement in improving firm performance, as all procurement practices are required, even if at varying degrees, for firm performance. This has far-reaching implications for procurement and supply chain practitioners. Accordingly, procurement practitioners should ensure that they provide these practices at their required levels to ensure firm performance. Practitioners should note that no extra performance improvements are attainable if investments are made into these procurement practices above the required levels.

Third, the findings underscore the importance of contract management and compliance and supplier partnership for achieving high firm performance. For some firms, high levels of contract management and compliance are required to ensure high levels of firm performance in two out of the three recipes that lead to high performance. Accordingly, firms should ensure high levels of contract management and compliance by investing heavily in initiatives and procedures that raise the levels of these practices to ensure high firm performance.

Fourth, these findings also highlight the critical role of supplier partnerships in ensuring high firm performance. For some of the cases, the presence (either low or high) of supplier partnership is required for firm performance in two out of the three recipes that lead to high firm performance. Whereas Model 1 requires the absence of supplier partnership in combination with high levels of compliance, high levels of supplier partnership in combination with high levels of both procurement planning and contract management is sufficient to guarantee high levels of firm performance in Model 3. Hence, firms should invest in building successful supplier partnerships as this is not an option but rather a requirement for high firm performance.

Fifth, the NCA results underscore the importance of procurement in ensuring value for money and firm performance. This finding portends important implications for managing the procurement and supply chain function. Moreover, procurement and supply chain managers should ensure that they make the investments that guarantee appropriate levels of these procurement practices to ensure firm performance. However, their investment in these practices should be only up to the levels that guarantee the maximum desired level of firm performance. This is because no additional increment in firm performance can be gained if the levels of investment in procurement are increased beyond the levels necessary to produce the highest level of performance.

### 6.3 Limitations and future research

The above-mentioned implications notwithstanding, the findings of this study are not without limitations and, hence, ought to be construed considering the following limitations. First, the paper explored the effects of procurement practices on the performance of private tertiary institutions in Ghana. However, the sample is considered homogenous in that they form one industry in a single country. Future studies should, therefore, expand the analysis using a more heterogeneous sample made up of firms from different sectors and countries. Second, the study used cross-sectional data called within a fixed time frame, hence could not account for temporary changes in the effect of procurement on firm performance. Future scholars should employ a longitudinal research design to identify and account for these temporary changes. Third, this study complements fsQCA with NCA by using fsQCA to identify complex configurations of procurement practices for firm performance and NCA to determine the levels of these procurement practices that are necessary for desired levels of firm performance. Hence it did not assess the distinct effects of procurement practices on firm performance. Future research should only use structural equation modelling to examine the individual effects of these practices on firm performance but also complement the chosen regressing-based analysis tool with either NCA or fsQCA or both to assess how procurement influences firm performance. Fourth, though this study operationalised procurement to consist of four key dimensions, namely, procurement planning, contract management, supplier partnership, and compliance, other practices may be included in the model to make it more comprehensive. Hence, future studies could consider appraisal and supplier development as either additional drivers or mediators of the relationships investigated in this study. Finally, the findings of this study are limited to the opinions of procurement professionals in private universities in Ghana; hence, it cannot be extended to other sectors of the Ghanaian economy. Future studies could consider other sectors, such as the automobile and auto parts industry or the manufacturing sector.

### 6.4 Conclusion

In contrast with prior studies on procurement and firm performance, which were largely based on symmetric thinking, this study sought to assess the asymmetrical link between procurement practices and firm performance. Hence, this study drew on complexity theory to answer the main research question of “What configurations of procurement practices lead to high levels of firm performance?” this study complemented fsQCA with NCA to determine the recipes of procurement practices that lead to both high and low levels of firm performance. These techniques permitted the identification of not only the necessary procurement for firm performance but also the identification of the practices that are required for firm performance besides the coveted levels of necessity. The results of fsQCA and NCA enhanced our appreciation of how procurement practices combine to produce different levels (high and low) of firm performance. Accordingly, the finding of this study does not only contribute to the emerging debate on how procurement contributes to firm performance but also expands the discourse regarding the link between procurement practices and firm performance amongst private tertiary institutions.

## Data availability statement

The authors do not have permission to share data.

## CRediT authorship contribution statement

**Innocent Senyo Kwasi Acquah:** Writing – review & editing, Writing – original draft, Visualization, Resources, Project administration, Methodology, Investigation, Formal analysis, Data curation, Conceptualization.

## Declaration of competing interest

The authors declare that they have no known competing financial interests or personal relationships that could have appeared to influence the work reported in this paper.

## Figures and Tables

**Table:** 

Criteria	CP	CM	PP	SP	FP
Cronbach's Alpha (CA)	0.823	0.868	0.916	0.764	0.926
Composite Reliability (CR)	0.887	0.900	0.930	0.864	0.937
Average Variance Extracted (AVE)	0.675	0.601	0.596	0.680	0.579
Variance Inflation Factor (VIF)	2.011	2.174	2.238	2.002	
**Construct**					
	**1**	**2**	**3**	**4**	**5**
1. Compliance (CP)					
2. Contract Management (CM)	**0.654** [0.524; 0.764]				
3. Firm Performance (FP)	**0.624** [0.458; 0.765]	**0.709** [0.564; 0.808]			
4. Procurement Planning (PP)	**0.784** [0.657; 0.885]	**0.702** [0.588; 0.789]	**0.611** [0.464; 0.732]		
5. Supplier Performance (SP)	**0.713** [0.553; 0.854]	**0.794** [0.688, 0.880]	**0.818** [0.711; 0.914]	**0.689** [0.545; 0.821]	

Figures in bold are HTMT values while those in paranthesis are 95% bias corrected and accelerated confidence intervals.

**Table:** 

PP	CM	SP	CP	Number	FP	Raw consist.	PRI consist.	SYM consist
1	1	1	1	19	1	0.912	0.825	0.830
1	0	0	1	5	1	0.910	0.448	0.481
1	1	0	1	2	1	0.892	0.374	0.386
0	0	0	1	4	1	0.830	0.372	0.397
0	1	1	1	14	1	0.829	0.514	0.594
0	1	0	1	2	1	0.825	0.328	0.328
1	1	1	0	2	1	0.808	0.414	0.432
0	0	1	1	3	0	0.794	0.293	0.343
1	0	0	0	2	0	0.743	0.155	0.159
0	0	0	0	21	0	0.414	0.018	0.018

Note(s): pp = procurement planning; cm = contract management; sp = supplier partnership; cp = compliance.

**Table:** 

Models from subsample for High Firm Performance		Raw Coverage	Unique Coverage	Consistency
1. cm*cp		0.715	0.416	0.808
2. ∼pp*∼sp*cp		0.347	0.048	0.786
Solution coverage:	0.763			
Solution consistency:	0.792			
**Models from subsample for low Firm Performance**				
3. ∼cm*∼sp*∼cp		0.514	0.048	0.963
4. cm*∼sp*cp		0.302	0.051	0.848
5. pp*cm*sp*∼cp		0.294	0.105	0.830
6. ∼pp*∼cm*∼sp		0.506	0.021	0.936
Solution coverage:	0.747			
Solution consistency:	0.854			

Note(s): pp = procurement planning; cm = contract management; sp = supplier partnership; cp = compliance.

## References

[1] Bag S., Dhamija P., Gupta S., Sivarajah U. (2021 Dec 10). Examining the role of procurement 4.0 towards remanufacturing operations and circular economy. Prod. Plann. Control.

[2] Kerdpitak C., Waiyawuththan P., Yen W.H., Chantranon S. (2022 Jun 5). Marketing mix strategy, procurement strategy, and eco-labeling strategy on concept enviropreneurial orientation to business performance of Thai pharmaceutical industry. J. Posit. Sch. Psychol..

[3] Tinali G.Z. (2021 Dec 14). The mediation effect of procurement competence on the relationship between practices and performance of the public sector procurement in Tanzania using higher-order constructs in SmartPLS. Orsea J..

[4] Stoll O., West S., Hennecke L. (2021 Jun 26). Procurement of advanced services within the domain of servitization: preliminary results of a systematic literature review. Smart Serv. Summit: Digit. Enabler Smart Serv. Bus. Dev..

[5] Pisitsankkhakarn R., Vassanadumrongdee S. (2020 May 1). Enhancing purchase intention in circular economy: an empirical evidence of remanufactured automotive product in Thailand. Resour. Conserv. Recycl..

[6] Gu V.C., Zhou B., Cao Q., Adams J. (2021 Jul). Exploring the relationship between supplier development, big data analytics capability, and firm performance. Ann. Oper. Res..

[7] Callado-Muñoz F.J., Hromcová J., Sanso-Navarro M., Utrero-González N., Vera-Cabello M. (2022 Feb 17). Firm performance in regulated markets: the case of Spanish defence industry. Defence Peace Econ..

[8] Munyimi T.F. (2019 Jan 1). The role of procurement quality controls in procurement performance in the energy sector in Zimbabwe. Cogent Eng..

[9] Ambekar S.S., Deshmukh U., Hudnurkar M. (2021 Jan 22). Impact of purchasing practices, supplier relationships and use of information technology on firm performance. Int. J. Innovat. Sci..

[10] Kumar N., Ganguly K.K. (2021 Jul 15). External diffusion of B2B e-procurement and firm financial performance: role of information transparency and supply chain coordination. J. Enterprise Inf. Manag..

[11] Scur G., Kunimura A.A., Chang J. (2022 Apr 19). Aligning corporate strategy with supplier selection. IEEE Eng. Manag. Rev..

[12] Hallikas J., Immonen M., Brax S. (2021 Jul 13). Digitalizing procurement: the impact of data analytics on supply chain performance. Supply Chain Manag.: Int. J..

[13] Etse D., McMurray A., Muenjohn N. (2021 Feb 4). The effect of regulation on sustainable procurement: organisational leadership and culture as mediators. J. Bus. Ethics.

[14] Kinuthia J.K., Amuhaya J. (2023 Oct 26). Sourcing strategies and organizational performance of muranga Co-operative creameries Kenya. J. Bus. Strat. Manag..

[15] Hanna A.S. (2016 Sep 1). Benchmark performance metrics for integrated project delivery. J. Construct. Eng. Manag..

[16] Singh P.K., Chan S.W. (2022 Jun 1). The impact of electronic procurement adoption on green procurement towards sustainable supply chain performance-evidence from Malaysian ISO organizations. J. Open Innov.: Technol. Mark. Complex..

[17] Acquah I.S., Issau K., Dei Mensah R., Vanderpuye F. (2023). When stakeholder orientations matter: modelling employee orientation, shareholder orientation and supply chain orientation as necessary and sufficient conditions for firm performance. Heliyon.

[18] Chepng’etich C. Strategic Procurement Practices and the Performance of Devolved Systems of Governance in Kenya (Doctoral dissertation, JKUAT-COHRED). http://localhost/xmlui/handle/123456789/5866,.

[19] Mazharul Islam M., Alharthi M. (2020 Dec 5). Relationships among ethical commitment, ethical climate, sustainable procurement practices, and SME performance: an PLS-SEM analysis. Sustainability.

[20] Acquah I.S.K. (2023 Jun 9). Modelling the importance of collaborative culture and its dimensions for supply chain collaboration: a necessary condition analysis. RAUSP Manag. J..

[21] Ragin C.C., Drass K.A., Davey S. (2006 Jun 15). Fuzzy-set/qualitative comparative analysis 2.0. Tucson, Arizona: Dep. Sociol. Univ. Arizona.

[22] Dul J. (2021).

[23] Dul J. (2016 Apr 1). Identifying single necessary conditions with NCA and fsQCA. J. Bus. Res..

[24] Kopplin C.S., Rösch S.F. (2021 Nov 1). Equifinal causes of sustainable clothing purchase behavior: an fsQCA analysis among generation Y. J. Retailing Consum. Serv..

[25] Xiong Q., Sun D. (2023 May). Influence analysis of green finance development impact on carbon emissions: an exploratory study based on fsQCA. Environ. Sci. Pollut. Control Ser..

[26] Woodside A.G., Nagy G., Megehee C.M. (2018 Jan 1). Applying complexity theory: a primer for identifying and modeling firm anomalies. J. Innov. Knowl..

[27] Scheuer C.L., Loughlin C., Woodside A.G. (2022). Can you always catch more flies with honey than with vinegar? Applying an asymmetric approach to transformational leadership research. J. Bus. Psychol..

[28] Chuah S.H., Aw E.C., Yee D. (2021 Oct 1). Unveiling the complexity of consumers' intention to use service robots: an fsQCA approach. Comput. Hum. Behav..

[29] Pappas I.O., Woodside A.G. (2021 Jun 1). Fuzzy-set qualitative comparative analysis (fsQCA): guidelines for research practice in information systems and marketing. Int. J. Inf. Manag..

[30] Lythberg B., Newth J., Woods C. (2021 Aug 4). Engaging complexity theory to explore partnership structures: Te Tiriti o Waitangi/The Treaty of Waitangi as a structural attractor for social innovation in Aotearoa-New Zealand. Soc. Enterprise J..

[31] Chaouali W., Hammami S.M., Veríssimo J.M., Harris L.C., El-Manstrly D., Woodside A.G. (2022 May 1). Customers who misbehave: identifying restaurant guests “acting out” via asymmetric case models. J. Retailing Consum. Serv..

[32] Miao M., Go I., Ikeda K., Numata H. (2022 Jan 2). Brand equity effects on financial performance in Japanese fashion market: applying complexity theory via fsQCA. J. Global Fash. Mark..

[33] Um T., Chung N., Stienmetz J. (2023 Apr). Factors affecting consumers' impulsive buying behavior in tourism Mobile commerce using SEM and fsQCA. J. Vacat. Mark..

[34] Acquah I.S.K., Naude M.J., Sendra-García J. (2021 May 1). Supply chain collaboration in the petroleum sector of an emerging economy: comparing results from symmetrical and asymmetrical approaches. Technol. Forecast. Soc. Change.

[35] Pappas I.O., Mikalef P., Giannakos M.N., Kourouthanassis P.E. (2019 Apr 9). Explaining user experience in mobile gaming applications: an fsQCA approach. Internet Res..

[36] Delgosha M.S., Saheb T., Hajiheydari N. (2021 Sep). Modelling the asymmetrical relationships between digitalisation and sustainable competitiveness: a cross-country configurational analysis. Inf. Syst. Front.

[37] Torres P., Godinho P. (2022 Jun). Levels of necessity of entrepreneurial ecosystems elements. Small Bus. Econ..

[38] Olya H.G., Mehran J. (2017 Jun 1). Modelling tourism expenditure using complexity theory. J. Bus. Res..

[39] Lou Z., Ye A., Mao J., Zhang C. (2022 Feb 1). Supplier selection, control mechanisms, and firm innovation: configuration analysis based on fsQCA. J. Bus. Res..

[40] Jovanovic J., Morschett D. (2022 Jul 1). Under which conditions do manufacturing companies choose FDI for service provision in foreign markets? An investigation using fsQCA. Ind. Market. Manag..

[41] Hayajneh J.A., Elayan M.B., Abdellatif M.A., Abubakar A.M. (2022 Feb 1). Impact of business analytics and π-shaped skills on innovative performance: findings from PLS-SEM and fsQCA. Technol. Soc..

[42] Li F., Aw E.C., Tan G.W., Cham T.H., Ooi K.B. (2022 Sep 1). The Eureka moment in understanding luxury brand purchases! A non-linear fsQCA-ANN approach. J. Retailing Consum. Serv..

[43] Schiele H. (2019).

[44] Arrigo E. (2020 Jan 9). Global sourcing in fast fashion retailers: sourcing locations and sustainability considerations. Sustainability.

[45] Oldman A., Tomkins C. (2018 Dec 17).

[46] Gatobu F.H. (2020 Apr). Influence of procurement process on the performance of public entities (A case study of Nairobi county government). Int. J. Acad. Res. Bus. Soc. Sci..

[47] Barbosa-Povoa A.P., Pinto J.M. (2020 Jan 4). Process supply chains: perspectives from academia and industry. Comput. Chem. Eng..

[48] Rasheli G.A. (2016 Aug 8). Procurement contract management in the local government authorities (LGAs) in Tanzania: a transaction cost approach. Int. J. Public Sect. Manag..

[49] Nash R.C., Schooner S.L., O'Brien-DeBakey K.R., Edwards V.J. (2021).

[50] Helmold M., Samara W. (2019).

[51] Chaudhuri A., Ghadge A., Gaudenzi B., Dani S. (2020 May 4). A conceptual framework for improving effectiveness of risk management in supply networks. Int. J. Logist. Manag..

[52] Chen Y., Chen I.J. (2019 Aug 21). Mixed sustainability motives, mixed results: the role of compliance and commitment in sustainable supply chain practices. Supply Chain Manag.: Int. J..

[53] Broadstock D.C., Matousek R., Meyer M., Tzeremes N.G. (2020 Oct 1). Does corporate social responsibility impact firms' innovation capacity? The indirect link between environmental & social governance implementation and innovation performance. J. Bus. Res..

[54] Gunningham N., Sinclair D. (2019 Jan 15). Regulatory pluralism: designing policy mixes for environmental protection. Environ. Law.

[55] Jepchumba N., Ismail N. (2015). Role of vendor managed inventory on supply chain performance in milk processing firms in Kenya: a case of New Kenya Cooperative Creameries. Int. Acad. J. Procure. Supply Chain Manag..

[56] Islami X. (2023 Dec 1). Lean manufacturing and firms' financial performance: the role of strategic supplier partnership and information sharing. Benchmark Int. J..

[57] Wang T.C., Shu M.H. (2022 Oct 1). Optimum design of generalized adaptive sampling plan for solid supplier-buyer purchasing partnership with yield-driven validation. Expert Syst. Appl..

[58] Kangogo RK. Strategic Partnerships And Performance Of KCB Group Ltd (Doctoral dissertation, University Of Nairobi). http://hdl.handle.net/11295/99077.

[59] Kasina NK. Strategic alignment as a source of competitive advantage at equity bank (K) Ltd (Doctoral dissertation). 10.1007/s10461-011-0065-1.

[60] Seo Y.S., Jo D.H. (2021 Jan 28). 2021 21st ACIS International Winter Conference on Software Engineering, Artificial Intelligence, Networking and Parallel/Distributed Computing (SNPD-Winter).

[61] Cho M., Bonn M.A., Giunipero L., Jaggi J.S. (2021 Apr 1). Supplier selection and partnerships: effects upon restaurant operational and strategic benefits and performance. Int. J. Hospit. Manag..

[62] Firdaus A.F., Madelan S., Saluy A.B. (2021). Supplier/partnership selection system Analysis based on analytic hierarchy method process in oil and gas drilling project (case study: pt. kmi). Int. J. Innov. Sci. Res. Technol..

[63] Soltani Z., Zareie B., Milani F.S., Navimipour N.J. (2018 Nov 1). The impact of the customer relationship management on the organization performance. J. High Technol. Manag. Res..

[64] Zhang Y., Khan U., Lee S., Salik M. (2019 Jan 18). The influence of management innovation and technological innovation on organization performance. A mediating role of sustainability. Sustainability.

[65] Aksar M., Hassan S., Kayani M., Khan S., Ahmed T. (2022). Cash holding and investment efficiency nexus for financially distressed firms: the moderating role of corporate governance. Manag. Sci. Lett..

[66] Syriopoulos T., Tsatsaronis M., Gorila M. (2022 Dec 1). The global cruise industry: financial performance evaluation. Res. Transport. Bus. Manag..

[67] Agbim K.C. (2019). Social networking and the family business performance: a conceptual consideration. J. Entrepren. Manag. Innov..

[68] Cardinaels E., van Veen-Dirks P.M. (2010 Aug 1). Financial versus non-financial information: the impact of information organization and presentation in a Balanced Scorecard. Account. Org. Soc..

[69] Teece D., Peteraf M., Leih S. (2016 Aug). Dynamic capabilities and organizational agility: risk, uncertainty, and strategy in the innovation economy. Calif. Manag. Rev..

[70] Van Greuning H., Bratanovic S.B. (2020 Jun 10).

[71] Ahmad S., Connolly C., Demirag I. (2020 Mar). A study of the operationalization of management controls in United Kingdom private finance initiative contracts. Publ. Adm..

[72] Arun J.S., Cuomo J., Gaur N. (2019 Jan 30).

[73] Alamgir F., Banerjee S.B. (2019 Feb). Contested compliance regimes in global production networks: insights from the Bangladesh garment industry. Hum. Relat..

[74] Zhou Y., Zhu S., He C. (2017 Feb 1). How do environmental regulations affect industrial dynamics? Evidence from China's pollution-intensive industries. Habitat Int..

[75] Sharma S., Modgil S. (2020 Jan 16). TQM, SCM and operational performance: an empirical study of Indian pharmaceutical industry. Bus. Process Manag. J..

[76] Thongrawd C., Ramanust S., Narakorn P., Seesupan T. (2020 Apr). Exploring the mediating role of supply chain flexibility and supply chain agility between supplier partnership, customer relationship management and competitive advantage. Int. J. Supply Chain Manag..

[77] Nenavani J., Jain R.K. (2022 Apr 15). Examining the impact of strategic supplier partnership, customer relationship and supply chain responsiveness on operational performance: the moderating effect of demand uncertainty. J. Bus. Ind. Market..

[78] Acquah I.S.K., Opoku Agyemang F., Baah C., Afum E., Agyabeng‐Mensah Y. (2023). Harnessing the value of procurement knowledge and practices for firm performance: a resource orchestration perspective. Knowl. Process Manag..

[79] Basheka B.C. (2008 Mar 1). Procurement planning and accountability of local government procurement systems in developing countries: evidence from Uganda. J. Public Procure..

[80] Changalima I.A., Mushi G.O., Mwaiseje S.S. (2021 May 7). Procurement planning as a strategic tool for public procurement effectiveness: experience from selected public procuring entities in Dodoma city, Tanzania. J. Public Procure..

[81] Hakiza J.B., Basheka B.C. (2012 Jan 1). Procurement planning and organisational conflicts in Kampala capital city authority-Uganda: implications for service delivery using a case of central division. Int. J. Procure. Manag..

[82] Matto M.C., Ame A.M., Nsimbila P.M. (2021). Influence of contract management on value for money procurement in Tanzania. Int. J. Procure. Manag..

[83] Oluka P.N., Basheka B.C. (2014 Jan 1). Determinants and constraints to effective procurement contract management in Uganda: a practitioner's perspective. Int. J. Logist. Syst. Manag..

[84] Srinivasan M., Mukherjee D., Gaur A.S. (2011 Aug 1). Buyer–supplier partnership quality and supply chain performance: moderating role of risks, and environmental uncertainty. Eur. Manag. J..

[85] Yeung K., Lee P.K., Yeung A.C., Cheng T.C. (2013 Aug 1). Supplier partnership and cost performance: the moderating roles of specific investments and environmental uncertainty. Int. J. Prod. Econ..

[86] Gallear D., Ghobadian A., He Q. (2015 Nov 2). The mediating effect of environmental and ethical behaviour on supply chain partnership decisions and management appreciation of supplier partnership risks. Int. J. Prod. Res..

[87] Richard P.J., Devinney T.M., Yip G.S., Johnson G. (2009 Jun). Measuring organizational performance: towards methodological best practice. J. Manag..

[88] Leiyan AP. Procurement practices and organizational performance: Case study of the University of Nairobi (Doctoral dissertation, University of Nairobi). http://hdl.handle.net/11295/98945.

[89] Jahanshahi A.A., Rezaei M., Nawaser K., Ranjbar V., Pitamber B.K. (2012 Jun 6). Analyzing the effects of electronic commerce on organizational performance: evidence from small and medium enterprises. Afr. J. Bus. Manag..

[90] Suárez C.A. (2016 Dec). Best management practices: SMEs' organizational performance management based on internal controls in Mexico. J. Int. Bus. Econ..

[91] Scarpi D., Confente I., Russo I. (2022 Nov 1). The impact of tourism on residents' intention to stay. A qualitative comparative analysis. Ann. Tourism Res..

[92] Pandey N., Kumar S., Post C., Goodell J.W., García-Ramos R. (2023 Sep). Board gender diversity and firm performance: a complexity theory perspective. Asia Pac. J. Manag..

[93] Rasoolimanesh S.M., Ringle C.M., Sarstedt M., Olya H. (2021 Jul 6). The combined use of symmetric and asymmetric approaches: partial least squares-structural equation modeling and fuzzy-set qualitative comparative analysis. Int. J. Contemp. Hospit. Manag..

[94] Ragin C.C. (2008). Evaluating set relations: consistency and coverage. Redesigning Soc. Inq.: Fuzzy Sets Beyond.

[95] Dul J., Van der Laan E., Kuik R. (2020 Apr). A statistical significance test for necessary condition analysis. Organ. Res. Methods.

[96] Vis B., Dul J. (2018 Nov). Analyzing relationships of necessity not just in kind but also in degree: complementing fsQCA with NCA. Socio. Methods Res..

[97] Shahjehan A., Afsar B., Shah S.I. (2019 Jan 22). Is organizational commitment-job satisfaction relationship necessary for organizational commitment-citizenship behavior relationships? A Meta-Analytical Necessary Condition Analysis. Econ. Res.-Ekon. Istraživanja.

[98] Sharma A., Dwivedi R., Mariani M.M., Islam T. (2022 Jul 1). Investigating the effect of advertising irritation on digital advertising effectiveness: a moderated mediation model. Technol. Forecast. Soc. Change.

[99] Du Y., Kim P.H. (2021 Jan 1). One size does not fit all: strategy configurations, complex environments, and new venture performance in emerging economies. J. Bus. Res..

[100] Eggers F., Risselada H., Niemand T., Robledo S. (2022 Jun 1). Referral campaigns for software startups: the impact of network characteristics on product adoption. J. Bus. Res..

