# Supplementary material for: Unravelling the asymmetric effects of procurement practices on firm performance: A complexity theory approach to complementing fsQCA with NCA
Source: Heliyon. 2024 Jan 29;10(3):e25230. doi: 10.1016/j.heliyon.2024.e25230 (PMC10850539; doi:10.1016/j.heliyon.2024.e25230)
Supplement: Multimedia component 1 [file mmc1.docx]

***Instruction: Kindly write or tick [√] where appropriate***

**Demographic Information**

1. Gender a. Male [ ] b. Female [ ]
2. Age range a. 20-30 years [ ] b. 31-40 years [ ] c. 41-50 years d. 51 years and above [ ]
3. Marital status a. Married [ ] b. Single [ ] c. Divorced [ ] d. Widowed [ ]
4. Educational background a. Diploma [ ] b. Degree [ ] c. Postgraduate [ ]

**Procurement Planning**

To what extent do you agree regarding the state and practice state of procurement planning within your institution?

| **No** | **Procurement planning procedure variables** | **1** | **2** | **3** | **4** | **5** | **6** | **7** |
| --- | --- | --- | --- | --- | --- | --- | --- | --- |
| 1 | Engaging in procurement planning activities |  |  |  |  |  |  |  |
| 2 | Adopting appropriate procurement planning format |  |  |  |  |  |  |  |
| 3 | Planning for procuring consultancy services |  |  |  |  |  |  |  |
| 4 | Planning for procuring technical services |  |  |  |  |  |  |  |
| 5 | Taking inputs from external stakeholders |  |  |  |  |  |  |  |
| 6 | Using inputs from the annual estimated budget for procurement planning |  |  |  |  |  |  |  |

***1=Very poor, 2=Poor, 3=Fair, 4=Good, 5=Very good, 6=Excellent and 7=Exceptional***

**Contract Management**

To what extent do you agree regarding the state and practice of contract management within your institution?

| **No** | **Contract management variables** | **1** | **2** | **3** | **4** | **5** | **6** | **7** |
| --- | --- | --- | --- | --- | --- | --- | --- | --- |
| 1 | Achieving the required quality service level |  |  |  |  |  |  |  |
| 2 | Ensuring compliance with the terms of the procured contract |  |  |  |  |  |  |  |
| 3 | Achieving low supplier defect rates |  |  |  |  |  |  |  |
| 4 | Following the due process in executing a contract |  |  |  |  |  |  |  |
| 5 | Communicating to all stakeholders in public contract |  |  |  |  |  |  |  |
| 6 | Communicating changes in contracts to all contracts |  |  |  |  |  |  |  |
| 7 | Maintaining good relationships with contracting parties |  |  |  |  |  |  |  |

***1=Very poor, 2=Poor, 3=Fair, 4=Good, 5=Very good, 6=Excellent and 7=Exceptional***

**Supplier Partnership**

To what extent do you agree regarding supplier partnership within your institution?

| **No** | **Supplier Partnership Variables** | **1** | **2** | **3** | **4** | **5** | **6** | **7** |
| --- | --- | --- | --- | --- | --- | --- | --- | --- |
| 1 | Monitoring materials and supplier |  |  |  |  |  |  |  |
| 2 | Information flows between organization and suppliers |  |  |  |  |  |  |  |
| 3 | Suppliers are included in continuous improvement programs |  |  |  |  |  |  |  |
| 4 | Main suppliers are part of planning and objective setting |  |  |  |  |  |  |  |

***1=Very poor, 2=Poor, 3=Fair, 4=Good, 5=Very good, 6=Excellent and 7=Exceptional***

**Compliance**

To what extent do you agree on compliance with procurement best practices in this institution?

| **No** | **Procurement best practice variables** | **1** | **2** | **3** | **4** | **5** | **6** | **7** |
| --- | --- | --- | --- | --- | --- | --- | --- | --- |
| 1 | Complying with specific organizational directives |  |  |  |  |  |  |  |
| 2 | Matching organizational rules with procurement regulations |  |  |  |  |  |  |  |
| 3 | Adhering to procurement rules without violating laid-down rules |  |  |  |  |  |  |  |
| 4 | Facilitating procurement objectives through available procurement rules |  |  |  |  |  |  |  |

**Organizational Performance**

To what extent do you agree regarding influence of best procurement practices on performance of this institution?

| **No** | **Organizational performance variables** | **1** | **2** | **3** | **4** | **5** | **6** | **7** |
| --- | --- | --- | --- | --- | --- | --- | --- | --- |
| 1 | Improved value for money |  |  |  |  |  |  |  |
| 2 | Promoting efficiency in procurement procedure |  |  |  |  |  |  |  |
| 3 | Avoiding unnecessary waste in procurement |  |  |  |  |  |  |  |
| 4 | Timely delivery of goods, services and works |  |  |  |  |  |  |  |
| 5 | Eliminating contracts breaches |  |  |  |  |  |  |  |
| 6 | Improving organizational efficiency |  |  |  |  |  |  |  |
| 7 | Enhancing timely response to customer needs |  |  |  |  |  |  |  |

***1=Very poor, 2=Poor, 3=Fair, 4=Good, 5=Very good, 6=Excellent and 7=Exceptional***
